# Community-engaged artificial intelligence research: A scoping review

**DOI:** 10.1371/journal.pdig.0000561

**Published:** 2024-08-23

**Authors:** Tyler J. Loftus, Jeremy A. Balch, Kenneth L. Abbott, Die Hu, Matthew M. Ruppert, Benjamin Shickel, Tezcan Ozrazgat-Baslanti, Philip A. Efron, Patrick J. Tighe, William R. Hogan, Parisa Rashidi, Michelle I. Cardel, Gilbert R. Upchurch, Azra Bihorac

**Affiliations:** 1 University of Florida Intelligent Clinical Care Center, Gainesville, Florida, United States of America; 2 Department of Surgery, University of Florida Health, Gainesville, Florida, United States of America; 3 Department of Medicine, University of Florida Health, Gainesville, Florida, United States of America; 4 College of Medicine, University of Central Florida, Orlando, Florida, United States of America; 5 Departments of Anesthesiology, Orthopedics, and Information Systems/Operations Management, University of Florida Health, Gainesville, Florida, United States of America; 6 Department of Health Outcomes & Biomedical Informatics, College of Medicine, University of Florida, Gainesville, Florida, United States of America; 7 Departments of Biomedical Engineering, Computer and Information Science and Engineering, and Electrical and Computer Engineering, University of Florida, Gainesville, Florida, United States of America; Harvard University, UNITED STATES OF AMERICA

## Abstract

The degree to which artificial intelligence healthcare research is informed by data and stakeholders from community settings has not been previously described. As communities are the principal location of healthcare delivery, engaging them could represent an important opportunity to improve scientific quality. This scoping review systematically maps what is known and unknown about community-engaged artificial intelligence research and identifies opportunities to optimize the generalizability of these applications through involvement of community stakeholders and data throughout model development, validation, and implementation. Embase, PubMed, and MEDLINE databases were searched for articles describing artificial intelligence or machine learning healthcare applications with community involvement in model development, validation, or implementation. Model architecture and performance, the nature of community engagement, and barriers or facilitators to community engagement were reported according to PRISMA extension for Scoping Reviews guidelines. Of approximately 10,880 articles describing artificial intelligence healthcare applications, 21 (0.2%) described community involvement. All articles derived data from community settings, most commonly by leveraging existing datasets and sources that included community subjects, and often bolstered by internet-based data acquisition and subject recruitment. Only one article described inclusion of community stakeholders in designing an application–a natural language processing model that detected cases of likely child abuse with 90% accuracy using harmonized electronic health record notes from both hospital and community practice settings. The primary barrier to including community-derived data was small sample sizes, which may have affected 11 of the 21 studies (53%), introducing substantial risk for overfitting that threatens generalizability. Community engagement in artificial intelligence healthcare application development, validation, or implementation is rare. As healthcare delivery occurs primarily in community settings, investigators should consider engaging community stakeholders in user-centered design, usability, and clinical implementation studies to optimize generalizability.

## Author summary

Most healthcare is delivered in community settings, but most healthcare research involving artificial intelligence is performed within academic or university hospital settings. There may be several reasons why most artificial intelligence research does not include data or perspectives from patients, providers, or administrators from community healthcare settings, but those reasons–and potential solutions to address them–are not well understood. Therefore, we systematically searched for relevant journal articles and used information from those articles to summarize the barriers and facilitators to community-engaged artificial intelligence research. We found that of approximately 10,880 articles describing artificial intelligence healthcare applications, only 21 (0.2%) described community involvement. Among those 21 articles, all derived data from community settings, but more than half used datasets that were probably too small for the model to perform at its best. Only one article described inclusion of community stakeholders in designing an application, which was accomplished by engaging community pediatricians and county Child Protective Services in designing an artificial intelligence model to detect child abuse in both hospital and community settings. As healthcare delivery occurs primarily in community settings, investigators should increase engagement with community stakeholders in designing and implementing artificial intelligence tools that serve everyone.

## Introduction

Artificial intelligence–computers performing tasks by mimicking human intelligence–is changing healthcare delivery [1]. By discovering complex, nonlinear associations, artificial intelligence algorithms often outperform simple additive models and rule-based inference engines [2,3]. To achieve equality in predictive performance, algorithms must be trained on datasets that accurately represent the patients to whom the algorithm will be applied; failure to meet this requirement risks performance degradation for rare cases and vulnerable populations [4,5].

Community-engaged research–which involves key stakeholders (e.g., patients, healthcare providers, administrators, and researchers) from community settings (here, community refers to settings outside academic hospitals)–should be allied with artificial intelligence research. Community engagement ensures that artificial intelligence tools are both generalizable to, and reproducible in, the most common site of healthcare delivery. Community engagement also has the potential to mitigate bias against underrepresented groups, which is already present in some AI tools. Academic centers performing artificial intelligence research may see patient populations that differ from those in surrounding communities; if those centers do not enroll patients whose socioeconomic and insurance status reflects the general public, dataset bias may result [6–11]. While training algorithms on datasets generated exclusively in academic centers could worsen healthcare disparities, community-engaged research could help to mitigate disparities by anchoring clinical decisions to accurate and objective predictions.

The degree to which contemporary artificial intelligence research involves community stakeholders has not been previously described, and could represent an important opportunity to improve scientific quality and the effectiveness of artificial intelligence-enabled tools, given its effectiveness in other domains [12,13]. This scoping review systematically maps what is known and unknown about community-engaged artificial intelligence research and identifies opportunities to optimize the generalizability of these applications through involvement of community stakeholders and data throughout model development, validation, and implementation.

## Materials and methods

Embase, PubMed, and MEDLINE databases were searched for articles describing artificial intelligence or machine learning with community involvement published between database inception and January 18^th^, 2023. Clinically-oriented databases of peer-reviewed articles were selected, rather than more technical, non-clinical databases, because this article focuses on healthcare applications that are intended for clinical audiences and clinical use, with the rationale that more technical, non-clinical article databases primarily contain development, validation, and theoretical work rather than community-engaged clinical research. Briefly, articles were included if they: 1) described the development or validation of an artificial intelligence healthcare application (e.g., algorithm, model, or artificial intelligence-enabled decision support tool), 2) described community involvement or engagement in the form of a) accruing data from community settings for algorithm training or testing, or b) inclusion of patients, providers, or administrators from community health care settings in user-centered design, usability, or clinical implementation studies, and 3) were published in English as a peer-reviewed journal article. Article search terms were as follows (note that “ab,ti” indicates presence of the search term in the abstract or title; * is a placeholder for any string of characters, such that the “engage*” term is fulfilled by “engaged,” “engagement,” “engage,” etc.): (community:ab,ti OR rural:ab,ti) AND (engage*:ab,ti OR involve*:ab,ti) AND (’artificial intelligence’:ab,ti OR ’machine learning’:ab,ti) AND [article]/lim AND [humans]/lim AND [english]/lim AND [clinical study]/lim AND ([embase]/lim OR [medline]/lim OR [pubmed-not-medline]/lim). All articles not meeting these criteria were excluded. The search terms identified 86 articles. After removal of duplicates, 45 articles remained. Exclusions at screening and full text review phases are illustrated in **S1 Fig**. Institutional Review Board approval and patient consent were not applicable to this review article.

Two reviewers independently screened abstracts for all 45 non-duplicated articles. Screening disagreements were resolved by a third reviewer via arbitration. The two screening reviewers had 78% agreement and a Cohen’s Kappa statistic for inter-rater reliability of 0.56, indicating moderate beyond-chance agreement [14]. Eighteen articles were excluded during the screening process because they did not meet inclusion criteria. For the remaining 27 articles, quality was rated using validated quality assessment tools [15]. Articles rated “poor” and those for which the full text did not meet inclusion criteria were excluded. Six articles were removed during full text review for not meeting inclusion criteria. Twenty-one articles remained and were included in the final analysis. Covidence software was used to organize article screening and selection as well as data extraction processes. Results were reported according to Preferred Reporting Items for Systematic Reviews and Meta-Analyses extension for Scoping Reviews (PRISMA-ScR) guidelines, as listed in **S1 Table**. Sources of funding and competing interests for each included article are listed in **S2 Table**.

For each included article, data extraction included the study population, artificial intelligence model architecture and predictive performance, whether model development or validation included data derived from community healthcare settings, whether community stakeholders (patients, providers, or administrators from community healthcare settings) were included in user-centered design, usability, or clinical implementation studies, and any barriers or facilitators to community engagement that were described within the article.

Finally, a separate search was performed to approximate the number of *all* articles describing artificial intelligence or machine learning healthcare applications, regardless of whether they reported community involvement or engagement, published between database inception and January 18^th^, 2023. The purpose of this search was to provide the denominator for calculating the proportion of artificial intelligence applications that describe community involvement. In this separate search, articles: 1) described the development or validation of an artificial intelligence healthcare application (e.g., algorithm, model, or artificial intelligence-enabled decision support tool) and 2) were published in English as a peer-reviewed journal article (i.e., mirroring the search for community-engaged artificial intelligence articles, but without the community engagement elements). This search identified 20,791 articles. Assuming this search yielded duplicates with a frequency that was comparable to the frequency of duplicates in the search for community-engaged artificial intelligence articles, there were approximately 10,880 non-duplicated articles describing artificial intelligence or machine learning healthcare applications.

## Results

### Approximate proportion of all artificial intelligence healthcare research with community engagement

**Fig 1:**
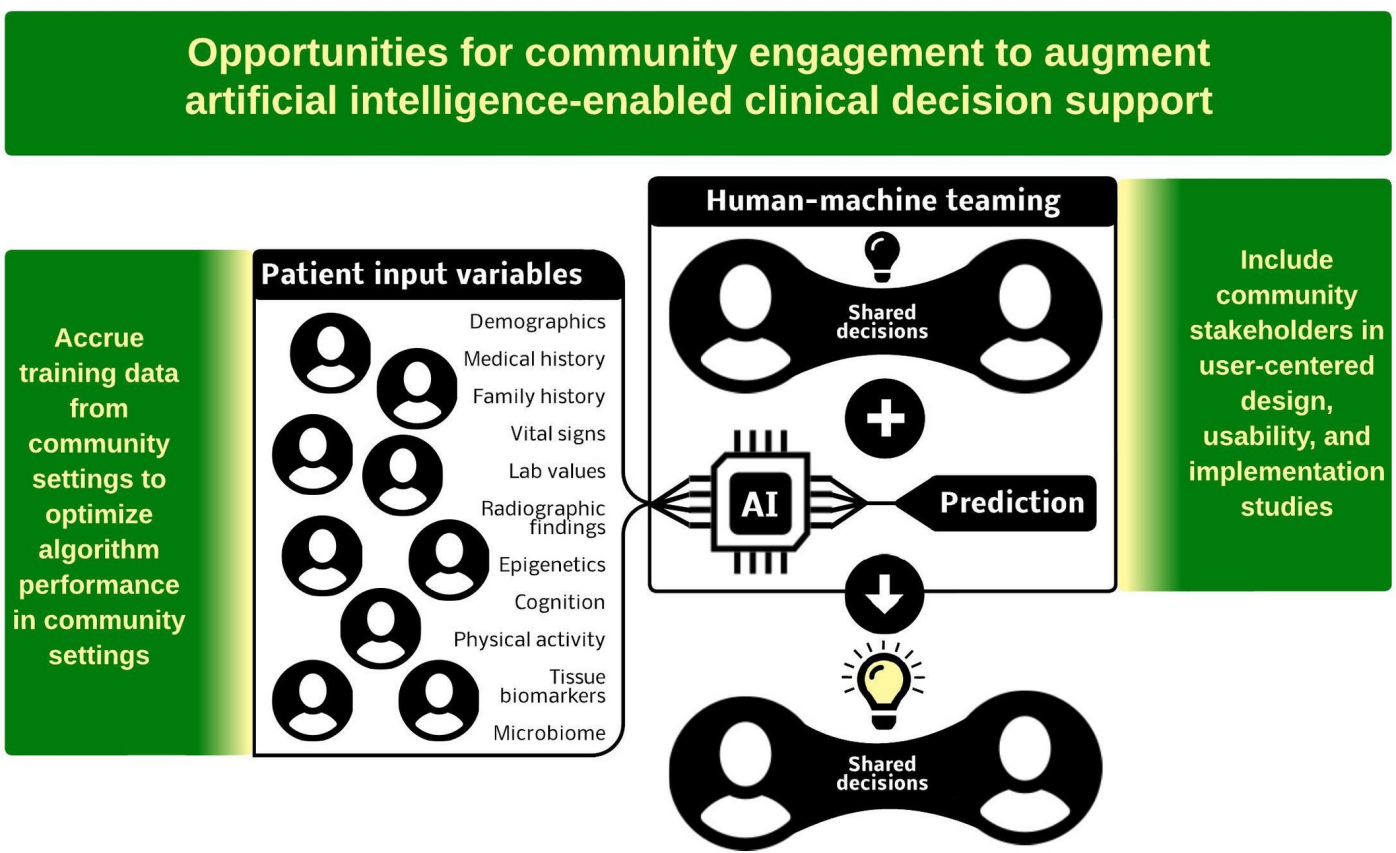
Community engagement themes and their role in artificial intelligence-enabled healthcare applications.

Based on the frequency of duplicates in the search for community-engaged artificial intelligence articles, we estimated that from inception to January 18^th^, 2023, Embase, PubMed, and MEDLINE databases contained approximately 10,880 non-duplicated articles describing artificial intelligence healthcare applications. Twenty-one of these articles (0.2%) described community-engaged artificial intelligence healthcare applications. All subsequent analyses refer to these 21 articles, which are summarized in **Table 1**. Eleven different countries are represented by the primary affiliations of the 21 first authors (Australia, China, Denmark, Germany, Ghana, India, Norway, Singapore, South Korea, Spain, and the United States of America). Community engagement themes and their role in artificial intelligence-enabled healthcare applications are illustrated in **Fig 1**.

**Table 1:** Summary of included studies.

Reference	Country of first author’s primary affiliation	Study population	Model architecture and performance summary^a^	Included community data	Included community stakeholders^b^	Description of community engagement	Barriers or facilitators to community engagement
Adua [16]	Ghana	219 patients with type II diabetes and 219 healthy individuals	A naive Bayes classifier had 87% accuracy and F1-score 0.87 for predicting type II diabetes	Yes	No	Healthy control participants were recruited from three local community suburbs	Convenience sampling facilitated recruitment of community participants, although this introduces sampling bias
Annapragada [17]	U.S.A.	867 pediatric patients	A bag of words natural language processing model detected cases of likely child abuse with accuracy 0.90±0.02 and AUROC 0.93±0.02	Yes	Yes	Referrals and framework design accounted for community hospitals rarely having adequate resources to maintain child abuse pediatrics teams	Harmonized electronic health records facilitated algorithm success in detecting abuse at both the main clinical location and satellite community referral sites
Astley [18]	U.S.A.	31,142,582 surveys from social media users in 114 countries/territories	A light gradient boosting machine predicted COVID-19 positive test results with AUROC 0.80 using self-reported symptoms and demographic data	Yes	No	Surveys were collected from social media users in 114 countries/territories	Social media survey dissemination facilitated a large, global sample for representing a global phenomenon, also imparting selection bias
Beevers [19]	U.S.A.	218 community adults with varying clinical levels of depression	A random forest model explained approximately 40% of the variance in depression symptoms	Yes	No	Community participants were recruited by oversampling individuals who are underrepresented in depression studies	None reported
Bharat [20]	Australia	41,787 survey respondents, of whom 791 had alcohol dependence by age 25 years	An ensemble model predicted alcohol dependence by age 25 years with AUROC 0.78 (0.74–0.81) and AUPRC 0.22	Yes	No	Respondents were enrolled from 14 community epidemiologic surveys in 13 countries	Survey collection was facilitated by leveraging an existing World Mental Health Survey Initiative
Brink-Kjaer [21]	Denmark	42 patients with isolated rapid-eye-movement sleep behavior disorder, 21 patients with other sleep disorders, 21 community controls	A model detected isolated rapid-eye-movement sleep behavior disorder with sensitivity 0.88 (0.79–0.94) and specificity and precision 1.00 (0.96–1.00)	Yes	No	Healthy control subjects were recruited from the community	Online advertisements facilitated recruitment of community healthy controls, which may impart selection bias
Caballero [22]	Spain	17,886 subjects age 50 years or greater participating in the English Longitudinal Study of Ageing	A random forest model had accuracy 0.83 in predicting ranges of health status scores	Yes	No	Data were obtained from community-dwelling adults	Community data collection was facilitated by using existing data from a UK Data Service catalogue available in an online repository
Clausen [23]	U.S.A.	301 adults with at least one psychological disorder, 276 health controls	An elastic net model predicted childhood trauma severity with root mean square error 0.23±0.01 and R^2^ 0.23±0.07	Yes	No	Subjects were recruited from community settings	Subject recruitment was performed through existing frameworks for nine previous or ongoing studies
Fukaya [24]	U.S.A.	493,519 individuals	A gradient boosting machine predicted varicose vein status with AUROC 0.70 using genotype data	Yes	No	Enrolled community participants	Enrollment of community participants was facilitated by use of the existing UK Biobank
Johannesen [25]	U.S.A.	20 subjects with schizophrenia and 12 healthy controls	A support vector machine model classified working memory tasks with 84% accuracy in healthy controls and 74% accuracy in subjects with schizophrenia	Yes	No	Enrolled healthy controls from the community	Despite offering $75 to participants a small sample was recruited, which is not ideal for machine learning models
Kim [26]	South Korea	650 participants age greater than 65 years living in rural South Korea	A random forest model predicted suicidal ideation with AUROC 0.78 (0.82–0.87) and positive predictive value 0.88	Yes	No	Participants were recruited from rural areas	Public health registries facilitated identification of eligible participants for recruitment into a convenience sample (650/2,839), introducing selection bias
Liu [27]	Singapore	973 adults who qualified as organ donors	A recursive partitioning model predicted organ donation decisions with accuracy 52% using participants organ donation fears and religion	Yes	No	Recruited subjects from community settings	Participants were recruited from communities door-to-door by simple random sampling of postal codes within districts during evenings/weekends and community eateries during lunch time
Moberget [28]	Norway	1,401 participants age 8–23	A shrinkage linear regression model predicted cognitive function with Pearson’s correlation of 0.20 using cerebellar morphometric features as predictors	Yes	No	Enrolled community participants	Enrollment of community participants was facilitated by using the publicly available Philadelphia Neurodevelopmental Cohort
Qian [29]	China	12,692 participants from rural China	An L1 regularized logistic regression predicted cardiovascular disease with AUROC 0.82 (0.80–0.83) using routine physical exam indicators	Yes	No	Enrolled community subjects	Stratified cluster random sampling was used to minimize selection bias in recruiting community subjects
Schwartz [30]	U.S.A.	247 community volunteers age 20–65 years	An elastic net model predicted sleep disorders with AUROC 0.80–0.85 using responses from a digital sleep questionnaire	Yes	No	Enrolled community subjects	Community subject enrollment was facilitated by Facebook advertisements, a convenience sample was selected, introducing selection bias
Shah [31]	U.S.A.	367 adults with spinal epidural abscess	An elastic net model predicted failure of non-operative management of spinal epidural abscess with AUROC 0.80 (0.76–0.83) using patient comorbidities, anatomic considerations, and physical exam findings	Yes	No	Patients were recruited from both academic and community hospitals	Identification of eligible community participants was facilitated by querying electronic records from both academic and community hospitals within the same hospital system
Tu [32]	Germany	11,365 urban students and 5,288 rural students from grades 1–3 across 17 cities in China	A gradient boosting decision tree predicted myopia with AUROC 0.92 using survey data and examinations of visual acuity, refraction, and routine eye examination	Yes	No	Enrolled community participants	A cluster stratified sampling method minimized selection bias by selecting two schools from each survey point and performing random sampling based on class
Walambe [33]	India	25 subjects that performed either traditional intelligence work or sedentary occupations	An artificial neural network had accuracy 0.97 and F1 score 0.95 in predicting stressed/not stressed states using posture, facial recognition, and computer interactions	Yes	No	Enrolled subjects from community environments	Enrollment of subjects from community environments was facilitated by use of a publicly available dataset
Yan [34]	U.S.A.	12,870 patients tested for COVID-19, 278 were positive, 208 were positive in outpatient/community settings	A model predicted a positive COVID-19 test with AUROC 0.78 (0.78–0.78) and AUPRC 0.30	Yes	No	Enrolled community subjects	Use of COVID-19 EHR DREAM challenge datasets allowed multiple teams to work simultaneously on a high-quality dataset
Zee [35]	China	945 subjects with diabetes and 1,276 community controls	A support vector machine model had sensitivity 1.00 and specificity 0.91 for predicting diabetes using retinal images	Yes	No	Enrolled community control subjects	Enrollment of community control subjects was facilitated by use of an existing community study of healthy individuals
Zhu [36]	U.S.A.	158,714 posts from Twitter profiles of state Medicaid agencies/managed care organizations	An ensemble classifier had accuracy 0.74 in predicting manually annotated labels of tweet content	Yes	No	Used public social media posts as the dataset	A public application programming interface was used to collect 158,714 public posts

^a^For the highest-performing or primary model. Performance metrics are presented with 95% confidence intervals in parentheses when available.

^b^Inclusion of community stakeholders (patients, providers, or administrators from community healthcare settings) in user-centered design, usability, or clinical implementation studies. U.S.A.: United States of America.

### Inclusion of data derived from community healthcare settings

All 21 studies included data derived from community healthcare settings. Six of the 21 studies (29%) compared cases or intervention group subjects with healthy control subjects. Of these six studies, 1 (17%) recruited all study subjects from community settings; [23] 4 (67%) recruited control subjects from community settings while recruiting cases or intervention group subjects from other clinical settings; [16,21,25,35] 1 (17%) recruited both cases and controls from community and non-community settings [34]. Fifteen of the 21 studies (71%) reported primary analyses of all study subjects as a single cohort. Of these 15 studies, 13 (87%) recruited all study subjects from community settings [18–21,24,26–30,32,33,36] and 2 (13%) recruited subjects from community and non-community settings [17,31].

### Inclusion of community stakeholders in user-centered design, usability, or clinical implementation studies

Only one study included community stakeholders in user-centered design, usability, or clinical implementation and deserves special mention. Annapragada et al. [17] developed a bag-of-words natural language processing model that detected cases of likely child abuse with accuracy 0.90±0.02 and area under the receiver operating characteristic curve (AUROC) 0.93±0.02. In addition to including cases from both hospital departments and smaller community settings, the prediction framework was developed with community engagement in mind. The authors note, “while large referral hospitals can maintain teams trained in Child Abuse Pediatrics (CAP), smaller community hospitals rarely have such resources, making the consistent detection of and response to subtle signs and symptoms of abuse difficult.” To offer similar protections for children both within and outside of large hospital settings, the authors trained and tested the prediction model on free text from pediatric electronic health records in both settings, using records from first contact to involvement of the child protection team. Community stakeholders included community pediatricians and county Child Protective Services. Although this study did not report user-centered design or usability experiments, it did include community stakeholders in developing a modeling approach used in implementation experiments, and was therefore classified as having stakeholder engagement.

### Facilitators and barriers to community engagement

The most common facilitator to including community-derived data was using an existing dataset that included community subjects. This approach was used in 6 of 21 studies (29%) [20,22,24,28,33,34]. The next most common facilitator was developing a novel dataset from existing data sources that included community subjects. This approach was used in 4 studies (19%) [17,18,31,36]. Internet-based publicly available sources were used for dataset generation or subject recruitment in 4 studies (19%) [18,21,30,36]. Convenience sampling was used in 3 studies (14%), which improved ease and efficiency but also introduced sampling bias [16,26,30]. To mitigate sampling bias, 3 studies (14%) used random or stratified sampling to identify representative subgroups of larger populations [27,29,32]. Subjects were recruited directly from other ongoing or completed studies in 2 studies (10%) [23,35]. Investigators traveled into communities (e.g., door-to-door or community restaurants) in 1 study (5%) [27].

The major barrier to performing community-engaged artificial intelligence research was small sample sizes. Eleven of 21 studies (53%) had overall sample sizes less than 2,000, risking overfitting [37,38] (i.e., learning associations or spurious correlations between inputs and outcomes that are not generalizable and rarely observed during external validation) [16,17,19,21,23,25,26,28,30,31,33]. Overfitting can be mitigated by regularization, cross-validation, and a reduction in model complexity. Overfitting is not always problematic, as some artificial intelligence models are intended for understanding associations within a study rather than producing generalizable knowledge. Despite this, small sample sizes may have affected more than half of all included studies. Additional challenges included sampling and selection bias being imparted by convenience sampling and surveys with low response rates, as well as a general lack of interoperability for deploying artificial intelligence tools in multiple environments without additional, special effort toward data harmonization.

## Discussion

The major finding from this study was that the incidence of community engagement in developing, validating, or implementing artificial intelligence applications was extraordinarily low. Almost all observed community engagement took the form of including data from community healthcare settings, with only one study explicitly included community stakeholders in user-centered design, usability, or clinical implementation. Most artificial intelligence applications focused on primary care, which typically involves longitudinal care provided in communities outside of hospital settings, which is conducive to community engagement relative to acute or emergency care, which typically involves intermittent care provided in hospitals. The most common facilitators of using community-derived data were leveraging an existing dataset that included community subjects or generating a novel dataset from sources that represent community subjects, especially using internet-based subject recruitment and data acquisition strategies. Many studies in which the investigators generated their own dataset had small sample sizes, risking overfitting. In addition, several studies performed convenience sampling or received low survey response rates, risking sampling and selection bias. As is often seen in contemporary analyses of artificial intelligence and digital health tools, we observed a general lack of interoperability.

We are unaware of any prior reviews on this topic. Although artificial intelligence modeling gained major performance advantages in 2012 and became prominent in healthcare literature over the ensuing decade, the maturation process of incorporating best practices from other fields–like community-engaged research–is ongoing [39]. We hope that our review will encourage community engagement in the future development, validation, and implementation of artificial intelligence healthcare applications.

Although there are inadequate examples in published literature to make evidence-based recommendations for best practices in community-engaged artificial intelligence, several potentially important themes emerge from this review. For model development, to obtain adequately sized training datasets that include community subjects, it seems advantageous to use large, existing datasets or harmonized electronic health record-derived data from multiple institutions [37,38,40–42]. Prospectively enrolling individual patients may be useful for validation and implementation studies, but resource requirements may preclude enrolling thousands of subjects during model development stages. Although not represented in the included studies, transfer learning (i.e., source models are trained on large datasets and then fine-tuned on smaller datasets of interest, like smaller community-derived datasets) could also address both sample size and generalizability issues [43–46]. Community stakeholders are an underutilized resource in model development and should be engaged early in any design process. Another potential strategy to promote engagement of community stakeholders is citizen science (i.e., scientific analysis of real-world data by members of the general public), which can expand the role of community members to active and equal members of research and technology development teams [47–49]. Each of these strategies has the potential to increase health equity by promoting the development, validation, and implementation of artificial intelligence tools that have all users in mind. Finally, community engagement should be encouraged in all healthcare application development, as it contributes to novelty and generalizability of the research product [50–53].

Despite relatively broad inclusion criteria, this study was limited by the small number of included studies. Although the small number of included studies could indicate that more time must pass before it would be appropriate to review community-engaged artificial intelligence healthcare applications, we see value in an early description of published work that highlights the paucity of evidence and identifies barriers and facilitators to future research. Knowledge of these themes may encourage investigators to accelerate the development, validation, and implementation of community-engaged artificial intelligence research. In addition, this review does not include more technical, non-clinical peer-reviewed journals, given the difficulty in replicating search parameters when surveying non-clinical bibliographic databases and because of our focus on intended clinical use.

## Conclusions

Community engagement in artificial intelligence healthcare application development, validation, and implementation is rare. Harmonized electronic health records from community care settings and large, existing datasets that include community subjects offer opportunities to train models on data that accurately represent community settings, without risk of overfitting and loss of generalizability. It may be advantageous to not only represent community subjects in model training, but also to engage community stakeholders–patients, providers, and administrators–in user-centered design, usability, or clinical implementation studies to ensure that artificial intelligence applications perform well not only in academic hospital settings, but also in community hospitals and clinics, where most healthcare is delivered.

## Supporting information

S1 Fig Article search parameters. (JPEG)

S1 Table Preferred Reporting Items for Systematic Reviews and Meta-Analyses extension for Scoping Reviews (PRISMA-ScR) checklist. (DOCX)

S2 Table Sources of funding and competing interests for included studies. (DOCX)
