# Supplementary figures and images for: Community-engaged artificial intelligence research: A scoping review
Source: PLOS Digit Health. 2024 Aug 23;3(8):e0000561. doi: 10.1371/journal.pdig.0000561 (PMC11343451; doi:10.1371/journal.pdig.0000561)

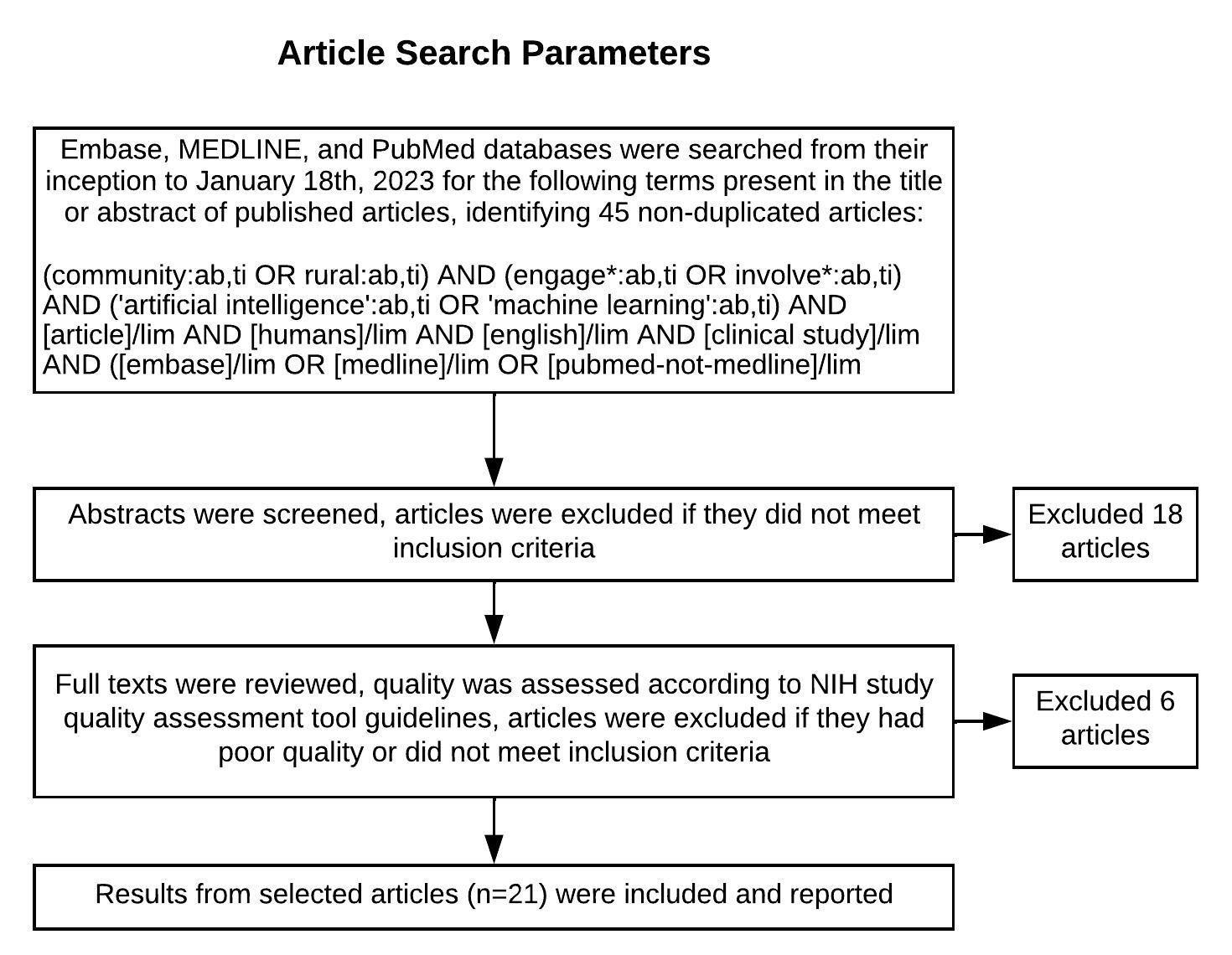

Supplement: S1 Fig — (JPEG) [file pdig.0000561.s001.jpeg]
