# Supplementary material for: Community-engaged artificial intelligence research: A scoping review
Source: PLOS Digit Health. 2024 Aug 23;3(8):e0000561. doi: 10.1371/journal.pdig.0000561 (PMC11343451; doi:10.1371/journal.pdig.0000561)
Supplement: S2 Table — (DOCX) [file pdig.0000561.s003.docx]

**S2 Table.** Sources of funding and competing interests for included studies.

| Reference | Sources of funding | Competing interests |
| --- | --- | --- |
| Adua^15^ | The study was supported by Edith Cowan University Collaboration Enhancement Scheme 2017. | None reported. |
| Annapragada^32^ | None reported | None reported |
| Astley^20^ | This work was supported by Facebook Sponsored Research Agreement INB1116217 and Facebook, Inc. Grant No. 4332732 | None reported |
| Beevers^21^ | Funding for this study was provided by National Institute of Health (awards R56MH108650, R21MH110758, R33MH109600) | None reported |
| Bharat^22^ | American Health Organization; Australian National Health and Medical Research Council; Bristol-Myers Squibb; Eli Lilly and Company; GlaxoSmithKline; John D. and Catherine T. MacArthur Foundation; National Institute of Health; National Institute of Mental Health; Ortho-McNeil Pharmaceutical Inc.; Pfizer Foundation; United States Public Health Service; UNSW Sydney | In the past 3 years, L.D. has received investigator-initiated untied educational grants for studies of opioid medications in Australia from Indivior, Mundipharma and Seqirus. M.G.H. reports consulting fees from RAND Corporation outside the submitted work. N.K. reports grants and consulting fees outside the submitted work. He received grants from Fujitsu Japan, Ltd and SBAtWork Corporation and consulting fees from Occupational Health Foundation, Japan Dental Association, Sekisui Chemicals, Junpukai Health Care Center and Osaka Chamber of Commerce and Industry. R.C.K. and N.A.S. report research grants from National Institute of Mental Health, USA(Grants: R01 MH070884; U01 MH60220); John D. and Catherine T. MacArthur Foundation; Pfizer Foundation; US Public Health Service (Grants: R13-MH066849, R01-MH069864 and R01DA016558); Fogarty International Center (Grant: R03-TW006481);Pan American Health Organization; Eli Lilly and Company; Ortho-McNeil Pharmaceutical; GlaxoSmithKline; Bristol-Myers Squibb; National Institute of Drug Abuse; Substance Abuse and Mental Health Services Administration, USA; Robert Wood Johnson Foundation (Grant 044708); and John W. Alden Trust. In the past3 years, R.C.K. was a consultant for Datastat, Inc., Holmusk, RallyPoint Networks, Inc. and Sage Therapeutics. He has stock options in Mirah, PYM and Roga Sciences. H.T. reports research grants from the Ministry of Health, Labour and Welfare, Japan. |
| Brink-Kjaer^16^ | E.D. was supported by the Feldman Foundation CA. A.B.-K. received funding for a research stay from the Stibo, OberstlÃ¸jtnant Max NÃ¸rgaard & Hustru Magda NÃ¸rgaards, OttoMÃ¸nsted, Augustinus, Knud HÃ¸jgaard, William Demant, Vera & Carl Johan Michaelsens, Tranes, Marie & M.B. Richters Fond, and IDAs &Berg-Nielsens Foundations. | E.M. was consul-tant for Jazz Pharmaceuticals, Sunovion, Avadel, Takeda, Eisai, Apple,Huami, Orexia, and Harmony. |
| Caballero^34^ | The ATHLOS project has received funding from the European Unionâ€™s Horizon 2020 research and innovation program under grant agreement No 635316. The first six ELSA waves have been funded jointly by UK government departments and the National Institute on Aging, in the USA. | None reported |
| Clausen^14^ | This work and all LIBR affiliates are supported by the William K. Warren Foundation, National Alliance for Research on Schizophrenia and Depression Young Investigator Grant (to W.K. Simmons), and National Institutes of Health (Grant No. K23MH108707 [to RLA], Grant No. K01MH096077 [to J. Savitz], and Grant No. K23MH112949 [to S.S. Khalsa]). Writing of the manuscript was partially supported by the Department of Veterans Affairs Office of Academic Affiliations Advanced Fellowship Program in Mental Illness Research and Treatment, Medical Research Service of the Durham VA Health Care System, and Department of Veterans Affairs Mid-Atlantic Mental Illness Research, Education, and Clinical Center. | W.K. Simmons is an employee of Janssen Research and Development, LLC, of Johnson and Johnson. |
| Fukaya^23^ | This study was performed with support from National Institutes of Health (1R01HL135313-01) and Knut and Alice Wallenberg Foundation (2013.0126) | Dr Lindholm reports institutional research grants from GlaxoSmithKline and AstraZeneca. Dr Ingelsson is a scientific advisor for Precision Wellness and Olink Proteomics. |
| Johannesen^17^ | Data collection and analyses were supported by a VA Rehabilitation Research & Development grants D7008W and 11603360 to JKJ. JB was supported by NSF grants IIS-1320586, NSF grant DBI-1356655, and NIH grant 1R01DA037349 during the study period. | None reported |
| Kim^24^ | Chungnam National University, Grant/Award Number: 2021-0754-01 | None reported |
| Liu^25^ | This work was supported by a grant awarded to CWL, TS, VKH, and JCJL from the National University of Singapore Humanities and Social Sciences research fund (grant number: HSS-1502-P02) | None reported |
| Moberget^26^ | This work was supported by the European Commission Seventh Framework Programme (Grant No. 602450 to IMAGEMEND), Research Council of Norway (Grant Nos. 213837, 223273, 229129, 204966/F20, 249795, and251134), South-Eastern Norway Regional Health Authority (Grant Nos.2013-123, 2014-097, 2015-073, 2016-083, and 2017112), and K.G. Jebsen Foundation. The Philadelphia Neurodevelopment Cohort sample is a publicly available data set. Support for the collection of the data sets was provided by the National Institute of Mental Health (Grant No.RC2MH089983 to Raquel Gur, M.D., Ph.D., and Grant No. RC2MH089924 to Hakon Hakonarson, M.D., Ph.D.). | None reported |
| Qian^27^ | This research was funded by the Non-profit Central Research Institute Fund of Chinese Academy of Medical Sciences (2020-PT330-003), the Shihezi University Innovation Outstanding Young Talents Program (Natural Science) (No. CXPY202004),and Shihezi University independently funded and supported school-level scientific research projects (No. ZZZC202018A). | None reported |
| Schwartz^28^ | This work was supported by dayZz Live Well Ltd (Herzliya,Israel). | Grant money and personal fees were provided by dayZz Live Well Ltd (Herzliya,Israel). |
| Shah^33^ | None reported | CB: Royalties: Wolters Kluwer (A), Elsevier (B); Consulting: UnitedHealth Care (B); Other Office: The Spine Journal (F); Fellowship Support: OMEGA (D, Paid directly to institution/employer).; JS: Scientific Advisory Board: Chordoma Foundation (None); Speaking and/or Teaching Arrangements: AO Spine (Travel Expense Reimbursement, Outside 12-Month Requirement), Stryker Spine (B, Outside 12-Month Requirement) |
| Tu^29^ | This research is funded by Hubei Provincial Central Committee Guides Local Science and Technology Develop-ment Special Project (No. 2019ZYYD058). It is also partially supported by the NSFC/DFG Collaborative Research Centre SFB/TRR169 "Crossmodal Learning" II | None reported |
| Walambe^30^ | This work was funded by the Symbiosis International University, Pune, India, under the research support fund. | None reported |
| Yan^19^ | This study was supported by the Clinical and Translational Science Awards Program National Center for Data to Health funding by the National Center for Advancing Translational Sciences at the National Institutes of Health (grant U24TR002306 [Ms Yan, Drs Schaffter, Bergquist, Guinney, and Mooney and Messrs Yu and Prosser]), Bill and Melinda Gates Foundation, the Institute for Translational Health Sciences (grant UL1 TR002319 [Dr Bergquist and Mooney and Mr Prosser]), and National Institutes of Health/National Institute of General Medical Sciences Anesthesiology and Perioperative Medicine Research Training (grant T32 GM086270 [Dr Long]). The CLEAR center was supported by the National Institute of Arthritis and Musculoskeletal and Skin Diseases (grant P30AR072572). | Ms Yan reported receiving grants from the National Center for Advancing Translational Sciences during the conduct of the study. Dr Bergquist reported receiving grants from National Institutes of Health and the Bill and Melinda Gates Foundation during the conduct of the study. Dr Brugere reported receiving personal fees from University of Illinois at Chicago, Salesforce, and Amazon outside the submitted work. Dr Long reported receiving grants from the National Institutes of Health/National Institute of General Medical Sciences during the conduct of the study. Dr Lee reported receiving grants from GE Healthcare to his institution; consulting fees for service on data safety monitoring board from GRAIL Inc; royalties from McGraw Hill Inc, Wolters Kluwer, and Oxford University Press; and payment for editorial board responsibilities for the Journal of the American College of Radiology from the American College of Radiology outside the submitted work. |
| Zee^18^ | This study was supported by the Hong Kong Innovation and Technology Fund - Midstream Research Programme (MRP/037/17X) | BZ and JL have a patent 'Method and device for retinal image analysis' licensed to Health View Bioanalytic, which received royalties through The Chinese University of Hong Kong. BZ and JL are founders and shareholders of Health View Bioanalytic, Bioanalytic Holdings, and Bioanalytic International Holdings. ML is the director of Bioanalytic Holdings and Bioanalytic International Holdings |
| Zhu^31^ | This study was supported by the Robert Wood Johnson Foundation (#76242). | None reported |
